# Micellar Composition Affects Lipid Accretion Kinetics in Molecular Dynamics Simulations: Support for Lipid Network Reproduction

**DOI:** 10.3390/life12070955

**Published:** 2022-06-24

**Authors:** Amit Kahana, Doron Lancet, Zoltan Palmai

**Affiliations:** Department of Molecular Genetics, Weizmann Institute of Science, Rehovot 761001, Israel; amit.kahana@weizmann.ac.il (A.K.); doron.lancet@weizmann.ac.il (D.L.)

**Keywords:** origin of life, Molecular Dynamics simulation, mixed micelles, accretion kinetics

## Abstract

Mixed lipid micelles were proposed to facilitate life through their documented growth dynamics and catalytic properties. Our previous research predicted that micellar self-reproduction involves catalyzed accretion of lipid molecules by the residing lipids, leading to compositional homeostasis. Here, we employ atomistic Molecular Dynamics simulations, beginning with 54 lipid monomers, tracking an entire course of micellar accretion. This was done to examine the self-assembly of variegated lipid clusters, allowing us to measure entry and exit rates of monomeric lipids into pre-micelles with different compositions and sizes. We observe considerable rate-modifications that depend on the assembly composition and scrutinize the underlying mechanisms as well as the energy contributions. Lastly, we describe the measured potential for compositional homeostasis in our simulated mixed micelles. This affirms the basis for micellar self-reproduction, with implications for the study of the origin of life.

## 1. Introduction

Lipids, sometimes regarded as amphiphiles or surfactants, are simple amphipathic molecules, possessing a polar headgroup and a hydrophobic tail. These molecules can spontaneously aggregate to form structurally diverse assemblies, such as micelles and vesicles, when present above certain threshold concentrations (e.g., critical micelle concentration, CMC). As the primary component of the membranes of all living cells, assemblies of lipids and their growth dynamics have been the focus of research for many decades [1]. This uncovered the technological usefulness of lipid assemblies, particularly in the fields of medicine and synthetic chemistry [2,3]. It has also offered insights on both the thermodynamic attributes of lipid assemblies, such as structural stability and electrostatics, and their kinetic parameters, such as their catalytic capacities [4,5].

As lipids are prebiotically available and possess capacities for spontaneous generation of supramolecular structures, these molecules have been widely implicated in studies of the origin of life. The traditional view of a protocell particularly invokes a vesicular bilayer surrounding an assortment of replicating polymers within its lumen interior [6,7]. The proposed role of the lipid bilayer in such studies is to sustain the replicating polymers and prevent them from diffusing away. This view has elicited a renewed focus on lipid systems in the context of prebiotic chemistry [8,9], typically comprised of simple lipids, such as fatty acids and alcohols [10]. Recent accounts [4,11] have suggested that lipids have other important functionalities besides acting as barriers between the protocellular inner network and the aqueous solution. The lipid membrane must be kinetically tied to the reproduction apparatus of the network, so that it will be able to coordinate its own growth and division with the entire cell [12,13]. This realization prompts a deeper exploration of the reproduction dynamics of non-covalent lipid assemblies as a significant facet of any origin-of-life scenario.

Going one step further, we have argued in previous studies [11,12] that mixed lipid assemblies may have facilitated the emergence of life without relying on replicating polymers. To test this Lipid First approach, we have developed the Graded Autocatalysis Replication Domain (GARD) chemical kinetics model, which directly illustrates how mutually catalytic interactions among lipids influence the accretion rates of new lipids joining a growing assembly. In this case, mutual-catalysis describes the predicted capacity of lipids to modify the rates of entry and exit of other lipid types to and from a micellar assembly, though synthetic reactions have also been explored in the context of the GARD model [11]. The model predicts that the micellar lipids act on accreting lipids in a coordinated fashion, as observed in published reports [12,14]. These catalytic exertions may lead to compositional homeostasis, a unique state in which the assembly’s composition remains unchanged throughout growth-and-split cycles [11,15]. Crucially, homeostatic growth followed by fission results in self-reproduction, mostly with mutations, which is a basis for subsequent selection and evolution. The inferences of the GARD model apply mostly to micelles rather than to vesicles, making them better life precursors [12].

According to the GARD model, the mutually catalytic interactions entail a kinetic influence of the momentary composition of a growing assembly on the accretion rates of joining monomers. More explicitly, the model assumes that each lipid type may influence the kinetics of any other lipid’s entry into or exit from the micelle. Thus, the micellar current constituency plays a critical role in determining monomer fluxes, hence the time-dependent changes of the micelle’s compositional states. It provides a recursive feedback loop, which induces deviations from random accretion. Monte Carlo simulations of the GARD model reveal that this guided progression allows an approach towards compositional homeostasis and self-reproduction [16,17].

While lipid-based non-covalent catalysis cases have been presented before [18,19,20], and some reports indicate that specific lipophiles may act as catalysts for the incorporation of new lipid monomers into vesicles [21,22], limited molecular details have been obtained on the precise mechanism and dynamics of lipids accretion into heterogeneous lipid assemblies. The reason for this is the technical challenge of monitoring picoseconds compositional changes within a population of nanoscopic mixed micelles, so as to be able to track the entire accretion trajectory of multiple assemblies. Without such technological capacity, experimentally studying catalysed accretion in the lab remains a daunting task.

However, advanced computational methodologies allow us to overcome some of these difficulties. Molecular Dynamics (MD) simulations is an effective and reliable computational tool, extensively validated and broadly accepted as an accurate emulation of real chemistry [23,24]. Since its invention, MD has matured enough to enable high-resolution scrutiny of complex molecular systems that are often inaccessible to experimentation [25]. This makes MD one of the best investigative tool for probing lipid-based kinetic phenomena in systems chemistry and protobiology arenas [26]. Indeed, MD has been used extensively to research micellar systems [27,28,29,30,31,32,33], deriving both structural and dynamical attributes. Though significant attention has been given to the process of self-assembly [27,29,34], most of the work on heterogeneous lipid systems was conducted largely without significant compositional variation.

In this work, we present an atomistic-resolution MD simulation study of the accretion of lipid monomers towards mixed micellar assemblies. We simulated binary mixtures of five lipid types in different ratios, observed their aggregation, and measured the influence of the micellar composition on the kinetics of monomer entry and exit. We report that compositional changes do, indeed, affect these rates, and, to various extents, for different lipid types, lending credence to the realism of network-like mutually-catalytic interactions among lipids. We describe cohesive aggregation profiles for discrete lipid combinations, and calculated the accretion flux of our mixed micelles, and determined that some may attain compositional homeostasis in non-random configurations, supporting predictions by the GARD model.

## 2. Materials and Methods

System set-up: The 3D models of Lauric Acid (LAU), Dodecyl Phosphocholine (DPC), Dodecyl Dimethyl Ammonio Propane Sulfonate (DAS), Dodecyl Dimethyl Amine-oxide (DDA), and Sodium Dodecyl Sulfate (SDS) were built using the Micelle/Membrane Builder facility of CHARMM-GUI [35]. The size of the system as well as the amount lipid monomers were originally based on a previous study by Abel et al. [36], using similar specifications to observe the self-assembly of DPC lipids concluding in a single micelle. Likewise, 54 lipid molecules were packed in random positions and orientations in a simulation box of 7.4 × 7.4 × 7.4 nm^3^ using the gmx insert-molecules tool of Gromacs 2020.2 (https://doi.org/10.5281/zenodo.3773801, accessed on 21 May 2020) [37] for each system. The molecules were in high concentrations (~220 mM), well above their CMC. The systems were solvated with TIP3P water molecules. Each box was replicated by periodic boundary conditions. One lipid (LAU) is neutral, and two others (DPC, DAS) are zwitterionic, hence, they bear zero net charges. The last two (SDS and DDA) bear net charges. Sodium counterions are introduced for the negatively charged SDS, and chloride counterions are introduced for the positively charged DDA. These counterions were added in random positions to achieve a neutral net charge of the systems. The minimization procedure and set-up of molecular dynamics (MD) simulations were performed with GROMACS 2020.2 program package, using the CHARMM-36 all-atom additive force field containing lipid parameters [38]. The real space summation of electrostatic interactions was truncated at 1.2 nm, and the Particle Mesh Ewald (PME) method was used to calculate the electrostatic interactions beyond 1.2 nm with a grid spacing of 0.12 nm and an interpolation order of 4. Van der Waals interactions were calculated using a cut-off of 1.2 nm. The solvated systems were energy-minimized to eliminate unfavorable positions. Harmonic positional restraints were applied on the lipid head group atoms—carboxyl oxygen for LAU, phosphate phosphorus for DPC, ammonium nitrogen for DAS, ammonium nitrogen for DDA, and sulfate sulfur for SDS—and tailgroup atoms—carbons at positions 4, 8, and 12—to achieve smooth minimization. 5000 steps of steepest descent algorithm were used, adopting harmonic force constants of 1000 kJmol^−1^nm^−2^ for the abovementioned lipid atoms. The minimized systems were equilibrated over 2 successive runs: 125 ps (NVT, 1fs time-step) and 200 ps (NPT, 2 fs time-step). To allow water molecules and ions to adjust around the lipids, harmonic restraints with a force constant of 1000 kJmol^−1^nm^−2^ were applied on the same lipid atoms, as in the case of the minimization step.

Production of MD trajectories: All-atom MD simulations were performed on a local High Performance Cluster (HPC) called Chemfarm, with nodes of 2 GPUs and 36 CPUs using the CHARMM-36 force field of GROMACS 2020.2 package. All simulations comprise trajectories of 50 ns, with atomic coordinates recorded every 2 ps. Two sets of triplicates were performed for binary systems (containing two types of lipids), whereas one set of triplicates was performed for each pure system (containing a single lipid-type). For each replica, new initial random velocities were generated, and for each set of replicas new initial atom positions were set as well. For the binary systems, five different concentration ratios were performed (Table 1).

The following MD protocols were used: the integration time step was 2 fs; the isobaric–isothermal (NPT) ensemble was employed; the pressure was set to 1 bar using isotropic coupling to the Parrinello–Rahman barostat with a time constant of 5 ps and an isothermal compressibility of 4.5 × 10^−5^ bar^−1^; the temperature was kept constant at 300 K using the Nosé–Hoover thermostat with a time constant of 1ps. Bonds with hydrogen atoms were constrained using the Linear Constraint Solver (LINCS).

Overall Simulation analyses: The MD trajectories were analyzed with tools included in the GROMACS 2020.2 package and by in-house Python scripts. Molecular clusters of lipids were calculated with the gmx clustsize tool of GROMACS using a cut-off of 0.24 nm (unless mentioned otherwise in the text), the largest distance to be considered in a cluster. The cut-off choice was based on the database of atomic van der Waals radii used by GROMACS [39]. The equilibrium minimum pairwise distance between two lipid molecules is the equilibrium nucleus–nucleus distance of two hydrogen atoms, i.e., 0.24 nm (twice the van der Waals radius of a hydrogen atom). Visualization of molecular conformations was made with PyMOL (The PyMOL Molecular Graphics System, Version 2.3.0 Schrödinger, LLC, New-York, US.) and VMD, version 1.9.4a43 (11 June 2020) [40]. Further analyses and graphs were generated using Python.

Β-matrix analysis: We took the slopes of all modulator-probe pairs and divided them by the basal exit rate coefficient of the probe. The basal rates were measured in pure mixtures (clusters of 100% probe type). We then multiplied the results by 100 to acquire the clear percentages of change in rate coefficients. For example, the exit rate coefficient of probe A from clusters of lipids A and B:

(1) $$k_{exit\;of\;A}=k_{A}\left(f_{B}\times \beta_{AB}+1\;\right)$$

where $k_{A}$ is the basal exit rate of probe A, $f_{B}$ is the fraction of modulator B within the cluster, and $\beta_{AB}$ is the value in the β-matrix that corresponds to the extent that modulator B modify the exit rate of probe A. The equation is accommodating of micelles that include more than two types, as exemplified in the compositional flux analysis.

Headgroup Interaction Prevalence (HIP) analysis: We took all the probe residence reactions and measured, at each time-step, the minimal distance of each of the probe’s headgroup moieties to other moiety types in the cluster. If the distance was equal or below 0.3 nm, we noted that the moieties interaction is present. For each reaction, we calculated the fraction of the residence time in which each possible probe-cluster moieties interaction occurs (HIP values). Then, we grouped the reactions into two groups based on the duration of their residence times—long residence group (above 10 ns) and short residence group (1–2 ns)—and calculated the average HIP value for every possible headgroup pair interactions within each group. Lastly, we calculated the fold changes in the HIP values between these two groups for each moiety pair interaction, as a ratio of the long residence group over the short residence group. T-tests were applied to observe the significance of the fold changes.

Dynamic Headgroup Interaction Prevalence (HIP) analysis: We first divided all reactions into two groups based on their involved clusters composition (below 50%, and above 50% lipid modulator levels). Then, we calculated the presence of a probe-cluster moiety interaction across all compatible reactions in a specific group and did so separately for each relevant time-step (for the first and last 0.5 ns of residence time). The HIP trends were smoothed with a sliding window of 10 ps. Later, we calculated the difference (shift) in the HIP plots between the modulator-rich and modulator-poor groups. We did so only for cross-interactions (between lipids of different types), since same-type interactions exist in clusters of many additional lipid types and, thus, are not applicable to this analysis.

Probe Orientation analysis: The orientation of a lipid is defined as the angle between two vectors—the lipid vector, that connects a prominent atom in the lipid’s headgroup and its terminal carbon, and the vector that connects the lipid headgroup and the geometrical center of the involved cluster. The prominent headgroups atoms are: “N” for DPC, “S” for SDS, “N” for DDA, “S” for DAS, and the carboxyl “C1” for LAU. The probe orientations were calculated for all the residence reactions from the exit analysis, and separately for each time-step within the first and last 0.5 ns. The plots were smoothed with a sliding window of 10 ps. Similar to the dynamic HIP analysis, the residence reactions were divided into two groups based on the involved cluster composition (below 50%, and above 50% lipid modulator levels). Afterwards, all compatible reactions within each group were averaged discreetly for each time-step, generating a typical orientation progression for the group. Lastly, we calculated the difference (shift) in the probe orientation plots between the modulator-rich and modulator-poor groups.

Compositional flux analysis: We generated all possible compositions from the five employed lipid types, with a resolution step of 5%. We then inserted each composition into a variation of the GARD kinetic equation below, and calculated its accretion flux, as well as the cosine similarity (H) between them. This similarity measure reveals the level of compositional homeostasis, as high similarity means the flux drives the micelle towards its current composition. A successful reproducer is defined as a composition that has an H value of 0.9 or above. Lastly, the typical reproducing composition was generated from averaging the compositions of the 10 best reproducers (those with the highest calculated H values).

(2) $$\frac{dn_{i}}{dt}=\left(C\times k_{entry,i}\times \left(1+\overset{N_{G}}{\underset{j=1}{\sum }}\left(f_{j}\times \beta_{entry,ij}\right)\right)\right)-\left(k_{exit,i}\times \left(1+\overset{N_{G}}{\underset{j=1}{\sum }}\left(f_{j}\times \beta_{exit,ij}\right)\right)\right)$$

In the equation: $n_{i}$ is the amount of lipid type *i*, $N_{G}$ is the number of lipid types in the system, *C* is the environmental concentration of lipid *i*, $k_{entry,i}$ and $k_{exit,i}$ are the basal entry and exit rates of lipid type i into and from a homogeneous cluster (100% lipid *i*), $f_{j}$ is the fraction of the composition that pertains to modulator lipid type *j*, and $\beta_{entry,ij}$ and $\beta_{exit,ij}$ are the rate modification parameters of modulator *j* over probe *i*, taken from the derived β-matrices in the results (see Section 3.2 and Section 3.3).

## 3. Results

### 3.1. Varied Lipid Combinations Elicit Discrete Dynamic Profiles of Self-Assembly

**Figure 1:**
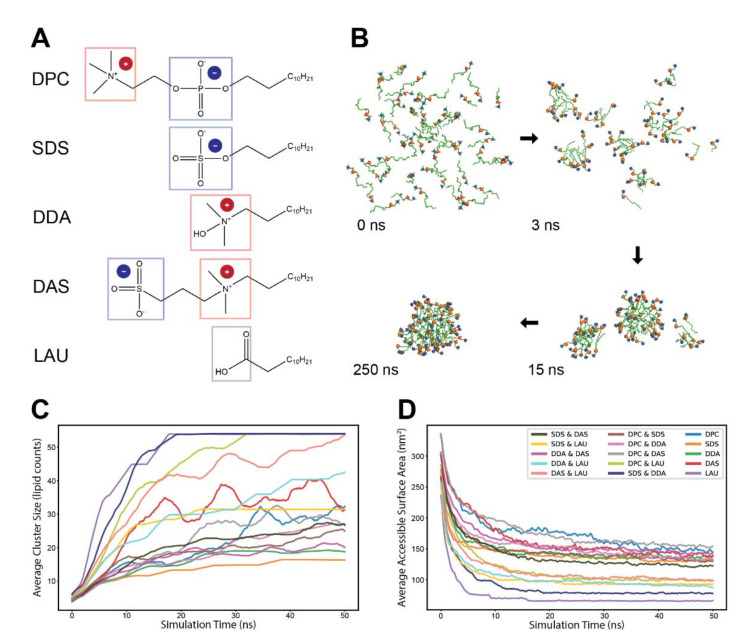
The self-assembly process of different lipid chemistries. (**A**) The molecular structures of the five lipid types employed in the simulations. The headgroup charged moieties are marked by color-coded frame and explicit charge indicator. (**B**) A typical self-assembly progression of lipids in an MD simulation. Depicted are 100% DPC molecules, with blue and orange spheres corresponding to headgroup moieties ammonium and phosphate, respectively. (**C**) Average size (lipid counts) of clusters in experiments of different lipid chemistries. Clusters start from size 2 and grow over the duration of the simulation. Line colors refer to different lipid chemistries (legend in (**D**)). The plots are an average of several simulations of either pure or mixed (50%/50%) lipid chemistries and were smoothed with a sliding window of 1 ns. (**D**) Average accessible surface area (SASA) of the employed lipids in the experiments. The plots are an average of several simulations of either pure or mixed (50%/50%) lipid chemistries, and were smoothed with a sliding window of 200 ps.

To test the effects of lipid compositions on accretion kinetics, we employed five widely different types of lipids, each with a distinct headgroup chemistry, so as to follow distinct self-assembly processes (Figure 1A). The lipids include two single-charge lipids (SDS, negative; and DDA, positive), two zwitterionic lipids (DPC, positive–negative; and DAS, negative–positive) and a neutral lipid (LAU). All lipids are reported to spontaneously aggregate to form micelles at equilibrium, except for LAU that mainly assembles into vesicles in a pure state [41]. All five lipids possess an identical hydrocarbon tailgroup of 12 carbons, allowing to focus solely on the effects attributed to headgroup chemistries.

For each experiment, we simulated 15 lipid combinations, 5 pure and 10 binary mixtures of the five lipid types at high concentrations (~220 mM) to facilitate fast accretion. Each run lasted 50 ns and consisted of 54 monomers randomly distributed in the simulation box, surrounded with explicit water molecules. The lipids spontaneously self-assembled into clusters, which grew bigger over time (Figure 1B). As observed before [33,42,43,44], two paths for cluster growth were present. While monomers are available in the environment (mostly during the first few nanoseconds), growth is driven primarily by stepwise addition of single monomers to existing small clusters. However, after monomers are practically depleted, the lipid clusters continued to grow mostly through fusion events, exhibiting much slower kinetics. Notably, general monomer depletion occurred as even the concentration of single monomers in the simulation box is similar to their CMC [45,46,47,48].

Following the accretion trajectories of different lipid admixtures, we probed their self-assembly profiles using two complementary measures. First, we examined the average non-monomeric cluster sizes along the simulations (Figure 1C). We observed that the average cluster size is very sensitive to charge distributions—the simulations that reached a single micelle state were those with more neutral net charge (such as pure LAU), while those that remained at the average aggregation number of about 20 are those with more pronounced net charges (such as pure SDS and pure DDA). Notably, some lipid mixtures displayed slow kinetics and did not reach an accretion plateau.

The second measure of accretion was changes in the Solvent Accessible Surface Area (SASA) of the simulated lipids over time, a measure of their compactness or, conversely, their accessibility for further interactions (Figure 1D). The SASA plots of the different chemistries start high, then decrease as lipids aggregate and become less exposed to water. Admittedly, this analysis is sensitive to the molecular size of the employed lipids, so that chemistries that involve bigger and bulkier lipids tend to display greater solvent accessibility. Accounting for this shift, the plots follow a similar trend to those displayed in the cluster size examination.

The two analyses provide quantitative information that portray cohesive aggregation profiles for distinct lipid chemistries. Interestingly, the accretion profiles of some lipid combinations are strikingly different from those of both pure constituents, as best exemplified by the complete self-assembly of SDS with DDA with prominent compactness, while each of them hardly manifests a full-sized micelle by the end of the simulation (Figure 1C,D). This may illustrate cooperative stabilizing interactions between the lipids, similar to those experimentally reported for SDS and C_12_TAB (sharing similar topology with DDA) [49], with kinetic implications.

### 3.2. Exit Rates of Lipids from Pre-Micelles Are Significantly Affected by Compositional Variation

Deriving association and dissociation kinetics from MD simulations is often nontrivial, and the chosen strategy changes depending on the explored molecular system [50,51,52,53,54]. Kinetics by classical MD simulations is mostly studied in ligand-receptor systems, where single-molecule experiments are performed on a sole receptor-ligand pair. This setting makes it computationally demanding to derive statistically correct association and dissociation rates [55]. In contrast, self-assembling lipid systems unlock a variety of cluster sizes, shapes, conformations, and compositions, yielding an abundance of association and dissociation events of free-monomers (ligands) with respect to a lipid cluster (receptor), facilitating the derivation of statistically reliable kinetic rates.

**Figure 2:**
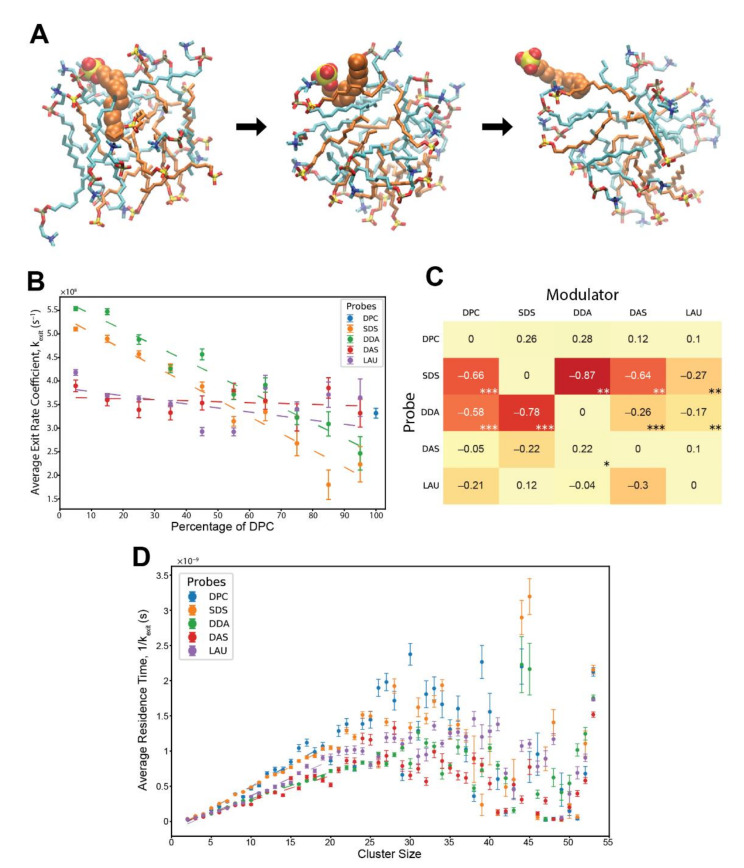
Exit reactions of monomeric lipids from pre-micellar clusters. (**A**) an SDS probe molecule escaping from a pre-micellar cluster. Orange—SDS, cyan—DPC. The probe is in space-filled representation, while the cluster lipids are in stick representation. The time from the first (fully inserted) to the last (dissociating) image is about 0.6 ns. (**B**) Kinetic analysis showing the average exit rate coefficients of different probe lipids influenced by varying levels of modulator DPC in the involved clusters. Error bars convey the standard error of the mean. R^2^ values for the weighted linear regressions: SDS—0.962, DDA—0.946, DAS—0.065, and LAU—0.286. The basal rate coefficient of DPC, at pure DPC clusters, is depicted for contrast. (**C**) The matrix of all composition-induced modifications to the exit rate coefficients. Values correspond to the extent of rate modification for each probe-modulator pair, in respect to the probe’s basal rate. Colors correspond to the magnitude of the values. Stars represent R^2^ values, whereby one star is 0.55–0.7, two stars are 0.7–0.85, and three stars are 0.85–1. (**D**) Average residence time of different lipid probes within clusters of various sizes. Error bars convey the standard error of the mean. R^2^ values for the weighted linear regressions, for clusters sizes 2 to 20 lipids: DPC—0.937, SDS—0.997, DDA—0.993, DAS—0.919, and LAU—0.976.

We adopted a reaction-based methodology for detecting instances of lipids associating with, and dissociating from, pre-micellar clusters. Such transitions were detected and recorded for all employed lipids from all the simulations, noting the composition and size of the involved clusters in each transition. Using this methodology, lipid exit rates were first measured (Figure 2A, Supplementary Video S1). This is, generally, a much simpler analytic measurement than that of entry rates, due to it being a first-order reaction. Based on previous ligand-receptor studies, we regarded the involved cluster as the receptor, its constituent lipid types that may influence the exit kinetics as “modulators”, and the leaving lipid as the “probe”. A “residence time” was defined as the duration between the first and last contact of the probe with the cluster, and its inverse is defined as the exit rate coefficient (Equation (3)). A similar definition of the term is used in the realm of ligand-receptor complexes [54,56,57,58,59,60,61], and has been discussed in the context of micellar systems as well [62,63]. In the analysis, we only inspected residence reactions that occurred for more than 1 ns in clusters of at least 10 monomers, to make sure the probe was fully inserted within a micellar phase before its expulsion.

(3) $$k_{exit}=\langle \frac{1}{Residence\;Time}\rangle$$

Figure 2B shows that with increasing levels of modulator DPC within the clusters, the exit rate coefficients of SDS and DDA drastically diminish, while those of DAS and LAU are unaffected. Interestingly, the measured rate coefficients are an order of magnitude higher than observed experimentally, for pure SDS and pure C_12_TAB micelles [64,65], explainable by the disordered nature of the pre-micelles as compared to fully-formed micelles in the lab, and, perhaps, also by the limitations of the computational model, such as the choice of the employed force-field.

By systematically mapping the kinetic modulation for all probe-modulator pairs, we generated a matrix of rate modifications (Figure 2C). This matrix represents an MD-derived estimate of β-matrix of mutually-catalytic interactions in the GARD model [16,66] (see Methods). The results clearly demonstrate that the most affected lipid probes are SDS and DDA, having their exit rates diminished by all lipid modulators with varying powers and linearity scores, while other lipids experience very mild catalysis. Notably, the highest kinetic influences in the matrix occur mutually between SDS and DDA, reflecting the cooperativity observed in their accretion profiles (Figure 1C,D).

Lastly, we examined the influence of cluster size on the probe residence times. For this, we combined all reactions of the same lipid probe type with all other lipid modulators and calculated typical residence times for all possible cluster sizes. The results depict linear ascents for all probes, ranging from cluster sizes of 2 monomers to about 20 monomers (Figure 2D). This phenomenon has been demonstrated before in vesicles [31], explained by a higher packing order of lipids, and was mostly absent in micelles of shorter lipids [44]. For larger cluster sizes, the residence times observed seem to peak between 25–35 monomers and diminish for even bigger clusters with higher observed variation. This trend could similarly be a result of greater molecular crowdedness in bigger clusters with increasing number of stabilizing hydrophobic interactions [31]. This data expands the previous analysis and demonstrates that variations in the micellar phase, either compositional or size-based, have substantial kinetic effects over exiting lipid monomers.

### 3.3. Entry Rates of Lipids into Pre-Micelles Show Sensitivity to Compositional Variation

**Figure 3:**
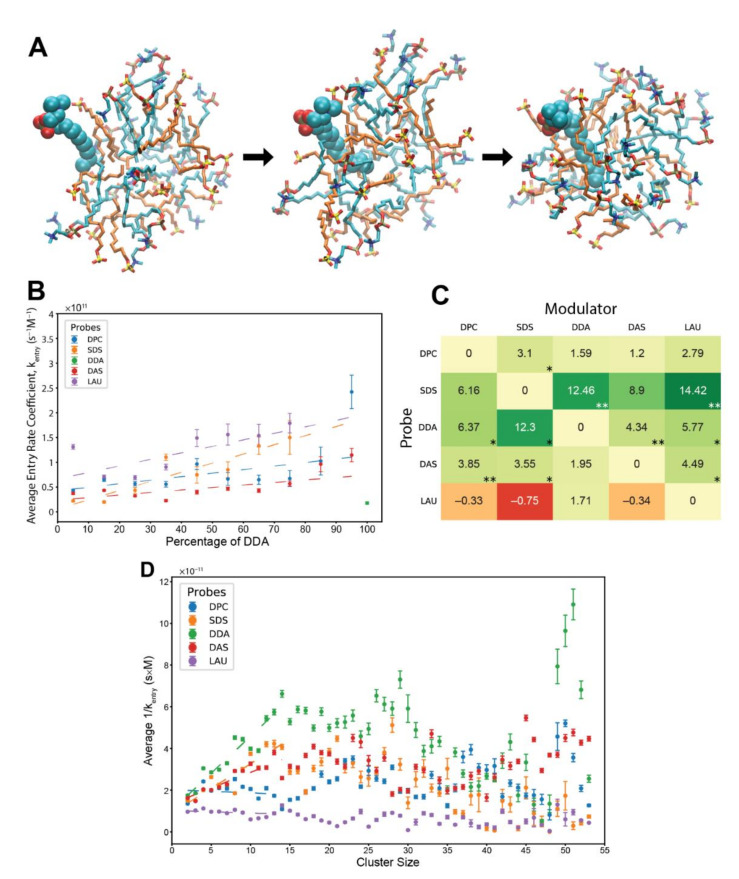
Entry reactions of monomeric lipids into pre-micellar clusters. (**A**) A DPC molecule joining a lipid cluster. Cyan—DPC, orange—SDS. The probe is in space-filled representation, while the cluster lipids are in stick representation. The time from the first (associating) to the last (fully-inserted) image is about 0.4 ns. (**B**) Kinetic analysis showing the average entry rate coefficients of different probe lipids influenced by varying levels of modulator DDA in the involved clusters. Error bars convey the standard error of the mean. R^2^ values for the weighted linear regressions: DPC—0.356, SDS—0.814, DAS—0.446, and LAU—0.481. The basal rate coefficient of DDA, at pure DDA clusters, is depicted for contrast. In the analysis, we eliminated a minority of cases in which the relative amount of the lipid components in the cluster changes by more than 5% of the total. We also subtracted from the addition time segments in which the concentration of free probes is zero and eliminated cases where the addition time is below 300 ps. (**C**) The matrix of all composition-induced modifications to the entry rate coefficients. Values represent the scope of rate modification for each probe-modulator pair, in respect to the probe’s basal rate. Colors correspond to the magnitude of the values. Stars represent R^2^ values, whereby one star is 0.55–0.7, two stars are 0.7–0.85. (**D**) Average residence time of different lipid probes within clusters of various sizes. Error bars convey the standard error of the mean. R^2^ values for the weighted linear regressions, for clusters sizes 2 to 14 lipids: DPC—0.020, SDS—0.834, DDA—0.890, DAS—0.732, and LAU—0.067.

In comparison to exit rates, the derivation of ligand entry rates in MD is not trivial, with multiple reported approaches optimized for different cases of protein-ligand binding [58,60,61,67]. When it comes to the explicit kinetic analyses of monomer entry into a lipid assembly (Figure 3A, Supplementary Video S2), the available literature is rather scant [33,44,51]. To accommodate this challenge, we adapted published algorithms in the realm of protein-ligand binding [54,57,58,59] to fit the lipid assembly system. We defined the term “addition time”, which is the duration between two consecutive entries of lipids of the same type. In this approach, the cluster into which the probe lipid enters includes the lipid that entered prior to it. Our defined “addition time” is analogous to the unbound time in ligand-protein systems [54,57,58,59], a duration in which a receptor is not bound to a ligand. It has been argued that the unbound time of a receptor, together with the concentration of free ligands, correspond to the entry rate coefficient. We therefore use the formula shown in Equation (4), whereby $C_{monomers}$ is the average of the varying concentration of free probe during the addition time. In the analysis, we only inspected residence reactions that occurred for more than 300 ps in clusters of at least 10 monomers.

(4) $$k_{entry}=\langle \frac{1}{Addition\;Time\times \langle C_{monomers}\rangle }\rangle$$

In general, the analysis presents rate coefficients that are about one order of magnitude faster than experimentally reported, for pure SDS and pure C_12_TAB systems [64,65]. Again, this may be a result of the limitations of the employed computational model, such as the choice of force-field, or likely the disordered nature of the simulated pre-micellar assemblies as compared to fully formed micelles in lab experiments. A known fact is that MD simulations can emulate relative differences of a measure better than its absolute values as experimentally reported [68]. Remarkably, the entry rate coefficients are also about 2–2.5 orders of magnitude higher than those of the exit rates, a difference that is very comparable to experimental reports [64,65]. Therefore, it seems that at the very least the results convey trends that are reliably similar to those of the actual rate coefficients.

The entry analysis shows that elevated levels of modulators in the involved clusters elicit mostly rough linear surges for the calculated rate coefficients (Figure 3B), with lower linearity scores than those that appear in the exit analysis. The detected mutually catalytic influences were summarized in accordance to the GARD concepts, in a similar manner to the β-matrix of the exit analysis (Figure 3C). Interestingly the entry matrix conveys similar trends to those that appear in the exit matrix, whereby the same modulator-probe pairs show significant rate modifications. This symmetrical pattern suggests that the same mechanism underlies both types of rate modification. It also accentuates the influence of cluster composition over accretion dynamics, as accelerated entry and decelerated exit both promote faster assembly growth.

Lastly, we investigated the influence of cluster sizes on the entry rate of lipids, independent of clusters composition (Figure 3D). We calculated the inverse of the entry rate coefficients for all lipid types across all relevant experiments and averaged them based on the size of their involved clusters. Some trends are similar to those of the exit analysis, such as linear ascents at small cluster sizes. However, the employed lipid types generally preserve the magnitudes of their kinetics across all cluster sizes, fitting previous results [44].

### 3.4. Specific Headgroup Interactions Contribute to Observed Rate Modifications

In addition to exploring the kinetic effects, it is important to demonstrate the molecular mechanisms that underlie this dynamic behavior. As the tailgroups of the employed lipids are nearly identical, these mechanisms likely involve only interactions between pairs of headgroup, neutral, charged, and zwitterionic (Figure 1A).

**Figure 4:**
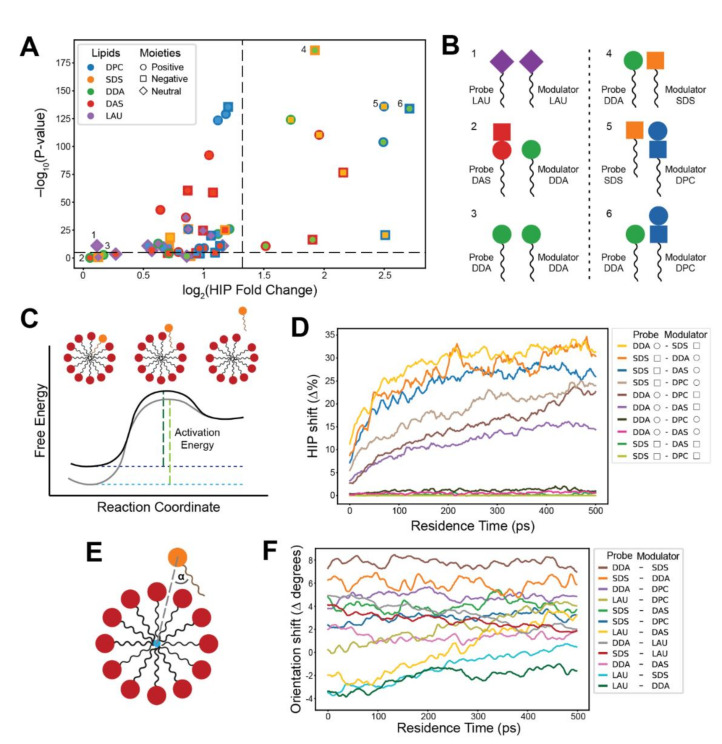
Specific headgroup interactions during lipid residence within clusters. (**A**) A volcano plot describing the fold change in Headgroup Interaction Prevalence (HIP) values between long residence (above 10 ns) and short residence (1–2 ns) reactions. Colors refer to lipid types; shapes refer to the charge of the interacting moiety. Inner shapes represent the probe’s moiety, and outer shapes represent moieties that belong to other lipids in the cluster. Black dashes indicate a 2.5-fold change and a 10^−5^ *p*-value. (**B**) Illustrations of representative lipid headgroup interactions with low (1–3) and high (4–6) HIP fold change. High HIP fold changes match the kinetic results, indicating that these interactions prolong probe residence times. (**C**) The energy landscape of lipid entry/exit transitions. Black corresponds to the basal energy profile of the transition, while gray corresponds to a profile modified by compositional variation as inferred from the observed kinetics. Green dashes represent activation energies for the exit reaction. (**D**) Dynamic HIP plots depicting the difference (shift) in HIP values between modulator-rich (50–100%) and modulator-poor (0–50%) clusters along the first 0.5 ns of the residence. Colors refer to distinct headgroup moieties interactions of probe-modulator pairs. Shapes in the legend refer to the charges of the moieties (see legend of (**A**)). Only interactions to the right of the vertical dashed line in (**A**) are included. (**E**) The probe orientation (α) in respect to the geometrical center of the cluster (blue circle). (**F**) A plot depicting the degrees shift in probe orientation in relation to the geometrical center of the involved clusters. The shift is between modulator-rich (50–100%) and modulator-poor (0–50%) clusters, along the first 0.5 ns of the probe residence. Positive shifts correspond to changes in orientation towards a tangential direction, while negative shifts correspond to changes towards a radial orientation. Colors refer to different probe-modulator pairs. Only single-moiety probes (SDS, DDA, and LAU) are included.

In order to determine the headgroup contributions, we further investigated the lipid-lipid interactions during probe residence reactions. We introduced a measure called Headgroup Interaction Prevalence (HIP), which is defined as the percentage of a reaction’s residence time in which a specific headgroup interaction is present. We further defined a HIP fold change, comparing two groups of reactions with high and low probe residence (see Methods). The results (Figure 4A) indicate that headgroup interactions play an important role in residence time elongation, since specific headgroup moiety interactions result in different HIP fold changes. The interactions that portray greater and more significant HIP fold changes match those exhibiting greater rate modifications in the exit kinetic measurements (Figure 2C), as illustrated in Figure 4B. Heterogeneous lipid headgroup interactions appear to contribute more to residence time elongation than homogeneous interactions, further emphasizing the power of lipid mutual catalysis [69]. Interestingly, for pairs of single-charge probes and cluster zwitterionic lipids, interactions with both zwitterionic headgroup moieties generally display substantial HIP fold changes, possibly due to the proximity of moieties on the lipid headgroup structure. Taken together, this analysis vividly displays the mechanistic interactions that underlie the observed compositionally driven kinetic effects.

The detected probe accretion kinetic effects indicate that the activation energy for exit and entry transitions is sensitive to the composition of the modulating clusters. The asymmetry between the exit rate decelerations (Figure 2C) and entry rate accelerations (Figure 3C) implies that variations in cluster composition may influence the energy levels of both the transition state and the micellar ground state (Figure 4C). In essence, it is possible that both catalysis and thermodynamic changes in affinity levels contribute to the observed kinetics. Validating these energy transformations for different micellar chemistries is a challenging task. Classical MD simulations have been used extensively to determine binding free energies for protein-ligand interactions, using a wide variety of methods for disparate chemical systems [24]. Self-assembling lipid systems that are dynamic and generate clusters of diverse sizes, compositions, and conformations may prove more tasking to dependably investigate as compared to proteins with more defined binding sites.

Therefore, we opted for a descriptive approach that focuses on changes in the mode of interaction of a probe with its modulating cluster. We devised two analyses that examine the lipid-cluster mode of interaction: a dynamic variation of the HIP analysis, and an analysis that follows the orientation of probes throughout their residence. While the first analysis focuses on molecular interactivity, the second investigates probe conformational transformations.

The dynamic HIP analysis shows the percentage of reactions in which a specific headgroup interaction is present at each time-step during the probe residence. We calculated these values for probes interacting with modulator-rich and modulator-poor clusters and plotted the difference (HIP shift) between these two groups (Figure 4D, Supplementary Figure S4A–D). The results indicate that elevated levels of modulator lipids in clusters indeed promote the majority of probe-modulator headgroup interactions, which is most prominent in chemistries that match substantial exit rate modifications. The analysis demonstrates how probes become engulfed in the cluster during their residence (Figure 4C,D), interacting selectively with neighboring lipids. The gradual changes in HIP shift throughout the residence, especially for kinetically affected chemistries (e.g., SDS–DDA), suggest that higher modulator concentration in clusters make these interactions more intensive within the pre-micellar cluster than in the transition states (at the beginning and end of the probe residence). Thus, it can be expected that the energy state of the probe within the cluster is more affected by compositional variation than that of the transition state.

However, there are indications that the transition state is somewhat affected as well. The association and dissociation phases of the residence show almost complete symmetry (Supplementary Figure S4A–D), pointing to cohesive modes of interaction. As HIP shifts are observed at the very beginning and end of the residence (i.e., the shallow depth of probe penetration into the cluster), these findings further strengthen the assertion that the modes of interaction for both monomeric entry and exit are highly similar with a definitive transition state [70] that is sensitive to compositional changes.

To further elucidate the lipid-cluster mode of interaction, a probe orientation analysis was introduced. It follows the orientation of the probe in regard to the geometric center of the involved cluster (Figure 4E). Similar to the dynamic HIP analysis, we calculated the shift in probe orientation (orientation shift) between modulator-rich and modulator-poor clusters (Figure 4F, Supplementary Figure S4E,F). As before, the most affected chemistries match probe-modulator pairs that experience substantial rate modifications, leading to more tangential probe orientations that suggest higher headgroup involvement.

The analysis depicts mostly no variation in the orientation shift along the residence, implying that increased modulator presence in clusters affects probe orientation equally at the transition state and at the bulk of the micellar residence. This is true except for chemistries involving LAU as probe or modulator. It appears that clusters with high LAU concentrations show more pronounced tangential orientations at the transition state, which continuously diminish throughout the probe residence. This behavior could again be clarified by the increased cluster compactness induced by the lipid (Figure 1C,D). High-LAU compactness could obligate probes to more forcibly disrupt the micelle during their transition and assume more tangential orientations. These changes may explain LAU-modulated detected kinetic effects. In sum, both dynamic analyses portray changes to lipid-cluster interaction modes, suggesting that although catalytic contributions are undeniably present, accretion kinetics are more influenced by compositionally driven affinity modifications.

### 3.5. Observed Accretion Kinetics Predicts Micelle Self-Reproduction at Non-Random Compositions

Self-reproduction of a chemical system is a widely accepted criterion for seeding life. As gleaned from the function of nowadays cells, a multicomponent chemical system must undergo homeostatic growth in which the relative concentration of each molecule in the system is preserved over growth and split cycles [11]. It has long been reported that micelles can grow through accretion of environmental lipids [43,64] or through endogenous synthesis [71,72], and can divide once they become too big and structurally unstable [73]. Some recent studies have provided experimental demonstration of compositional homeostasis in a population of proliferating mixed micelles [12,74,75], but detailed evidence at the single micelle level is still lacking.

From our MD simulations, we can see strong indication of compositional homeostasis. For example, we can look at the mutual interaction of SDS and DDA, accelerating the entry and decelerating the exit of each other with comparable strengths. If we account for only the exit rates (the more reliable of the two measures), we calculated—by taking the intersection point of the two curves of exit rates of SDS probe–DDA modulator and vice versa—that micelles with 45.25% DDA and 54.75% SDS will attain perfect compositional homeostasis, where both lipid types will display an identical exit flux. Other such pairs could display similar homeostatic states at different ratios.

**Figure 5:**
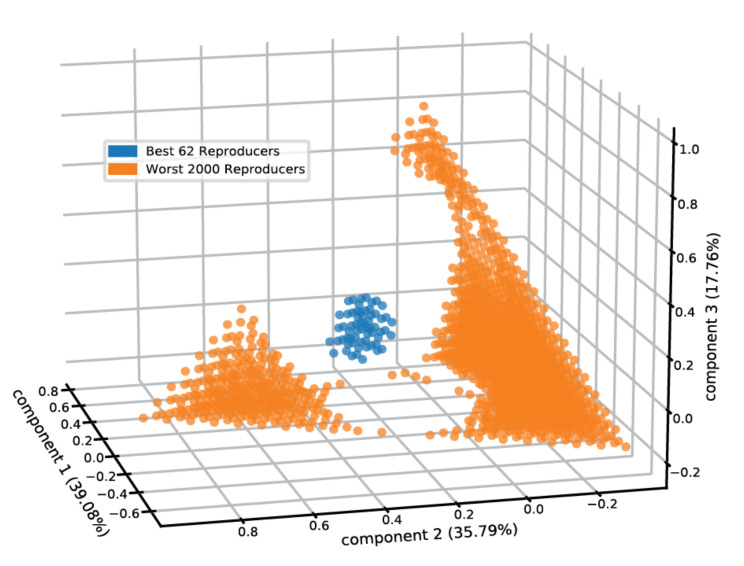
Micellar self-reproduction in five-dimensional compositional space. The PCA plot features the reproduction capacity of mixed micelles of different compositions containing the employed five lipids, within an environment where these lipids are in equimolar 4 mM environmental concentrations. The best 62 (0.58%) reproducers are shown in blue and the worst 2000 reproducers are in orange. We note that enhancement of the equimolar concentrations had negligible effects on the results.

Furthermore, acquiring the rate modification parameters by which one lipid influences the entry and exit rates of a second lipid allows us to tentatively examine the prospect of compositional homeostasis in more elaborate micellar clusters containing more than two lipid types. For that, we examined the compositional space of micelles comprised of the five employed lipid types (Figure 5) and calculated the entry and exit fluxes for the lipids (see Methods). Providing equimolar 4mM lipid concentrations in the environment, we observed that 0.58% of the compositional space “pixels” attains homeostasis. The centroid of the best self-reproducing assemblies contains 21% DPC, 36% SDS, 37.5% DDA, 3% DAS, and 2.5% LAU. The self-reproducing compositions are clustered together in space (Figure 5), matching the definition of a compotype (cluster of composomes, as predicted by the GARD formalism) [11].

An important aspect of the group of homeostatic compositions are how distant they are from the equimolar concentrations in the environment. This is a hallmark of the fact that these compositions are governed by kinetic phenomena away from equilibrium. Without composition-driven kinetic effects, whereby the entry and exit rates for all lipid types would be equal, equilibrium will be reached at compositions that reflect the outside concentrations [16]. The resultant homeostatic compositions in our analysis are more distant from the equimolar state than 40.38% of possible compositions. This nontrivial finding is significant as it portrays away-from-equilibrium dynamics for self-reproducing mixed micelles that defines living cells.

## 4. Discussion

The depicted MD results provide prime evidence that the temporary composition of pre-micellar assemblies affect their proceeding accretion trajectory in compositional space by modifying the entry and exit rates of aggregating lipids. Using simple analyses, we were able to quantify the mutual rate modifications among the simulated lipids, observing cooperative and selective interactions. We further described the mechanistic underpinnings of the observed kinetics and the contribution of lipid–micelle affinity. Finally, we extrapolated the kinetics of more elaborate mixed micelles based on the matrices of derived rate modification parameters, predicting the scope of compositional homeostasis in an emulated multi-component assembly. These results constitute an important step towards researching the origin of life in invoked simple lipid chemical systems.

Self-aggregation is a concerted interplay of monomers, primarily driven by hydrophobic interactions among tailgroup [76]. This insight is reflected in accretion kinetics, whereby changes to the length of lipid tailgroups could modify exit rates by several orders of magnitude [70,77,78]. This work uncovers the contribution of headgroup chemistry to the accretion kinetics, with some energetic influences. Previous reports illustrated the effects of headgroup chemistries on aggregate sizes and structures [76], and our work uncovers complementary key facets in lipid insertion and removal. Additionally, we observed a minor influence of cluster size on these kinetics, especially above monomer count ~30, possibly indicating that this trend is not dependent on system specifications (such as volume and monomer count).

Molecular Dynamics is an established experimental tool for investigating the emergent behavior and accretion kinetics in lipid systems [26], and has proven itself again in the present work, generating entry and exit rates that are highly comparable to experimentally measured rates [64,65]. Importantly, the observed kinetic trends match previous experimental reports, such as the cooperativity between single-charged positive and negative lipids [49] and the generally faster entry and slower exit kinetics of neutral lipids [79]. Therefore, we advocate that the MD results are expected to reliably display compositionally driven kinetic effects. Future work may improve our analytical endeavors, exploring more heterogeneous systems in longer and more elaborate simulations, and, perhaps, validating these results with advanced laboratory experiments.

One of the remarkable aspects of the kinetic analyses is the existence of certain mutual catalysis among different lipid types. This finding suggests that each lipid may act as a rate modifier and influence the accretion rates of another lipid in a micellar context. Mutual catalysis among simple molecules has been reported before [80,81], particularly in lipid systems [12,74], yet has not been quantitatively explored in detail. Excitingly, rate modifications appear to distribute across a broad scale, and, when present, they occur with significant probe-modulator specificity. These conclusions match previous GARD predictions [66,82], and strengthen the treatment of mixed micelles as nanoreactors [4,12]. It is reasonable to assume that mutual rate modification applies for most (if not all) lipophilic molecules, to various degrees, and, thus, more diverse micellar systems will produce more complex catalytic networks.

Lastly, the acquired results promote the validity of micellar self-reproduction. Affirming previous work [12], we present here the realism of compositional homeostasis in proliferating micelles, based on the derived rate modification parameters. This finding is highly significant in the context of the origin of life, providing, perhaps for the first time, direct evidence that growing micellar assemblies could truly self-reproduce, bequeathing their compositional information to progeny. Therefore, this significantly supports the possibility of life’s emergence in catalytic mixed micelles, paving a path for selection and evolution towards life as we know it.

## Figures and Tables

**Table 1:** Five different concentration ratios used for the binary systems.

	Ratio	Monomer Count
1	10%/90%	6/48
2	30%/70%	16/38
3	50%/50%	27/27
4	70%/30%	38/16
5	90%/10%	48/6

## Data Availability

Not applicable.

## Supplementary Materials

The following supporting information can be downloaded at: https://www.mdpi.com/article/10.3390/life12070955/s1.
