# Supplementary material for: Micellar Composition Affects Lipid Accretion Kinetics in Molecular Dynamics Simulations: Support for Lipid Network Reproduction
Source: Life (Basel). 2022 Jun 24;12(7):955. doi: 10.3390/life12070955 (PMC9325298; doi:10.3390/life12070955)
Supplement: Supplementary file 1 [file life-12-00955-s001.zip › Pap429_supplementary_figures.pdf]

Supplementary Figures

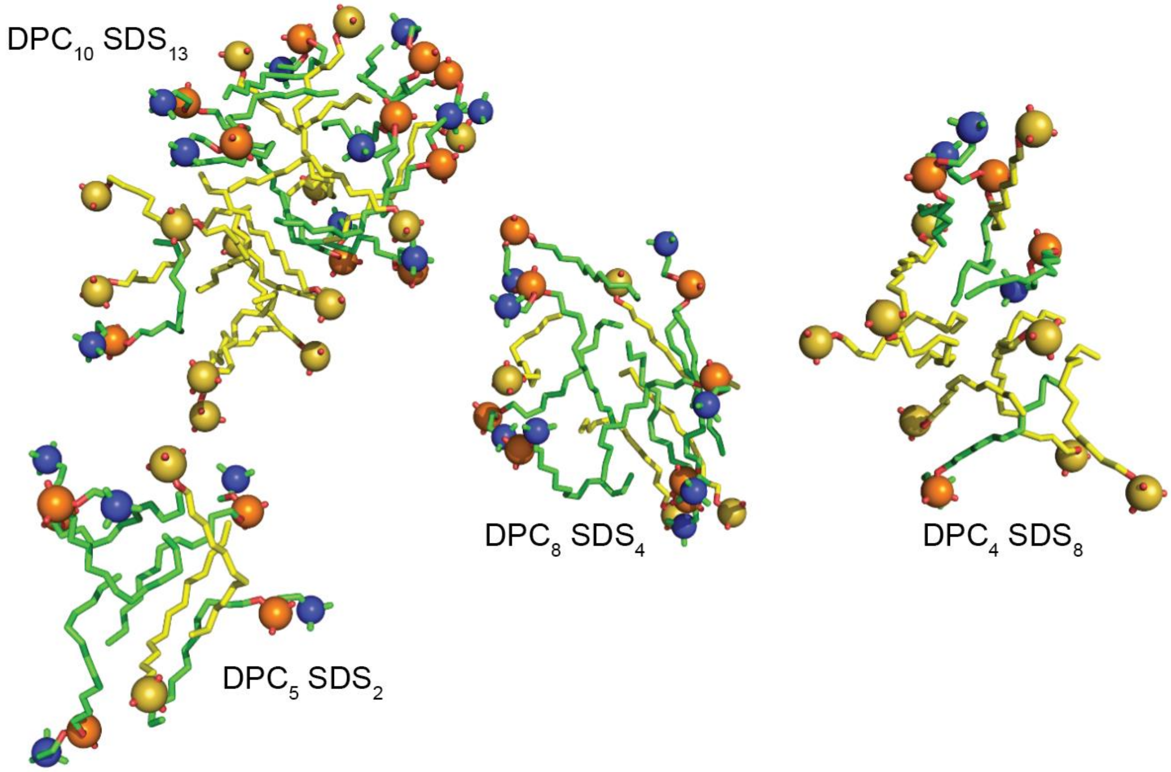

Supplementary Figure S1 – a typical frame from the MD simulations, depicting variability in cluster size and composition. Green - DPC, yellow – SDS. Spherical balls represent different headgroup moieties.

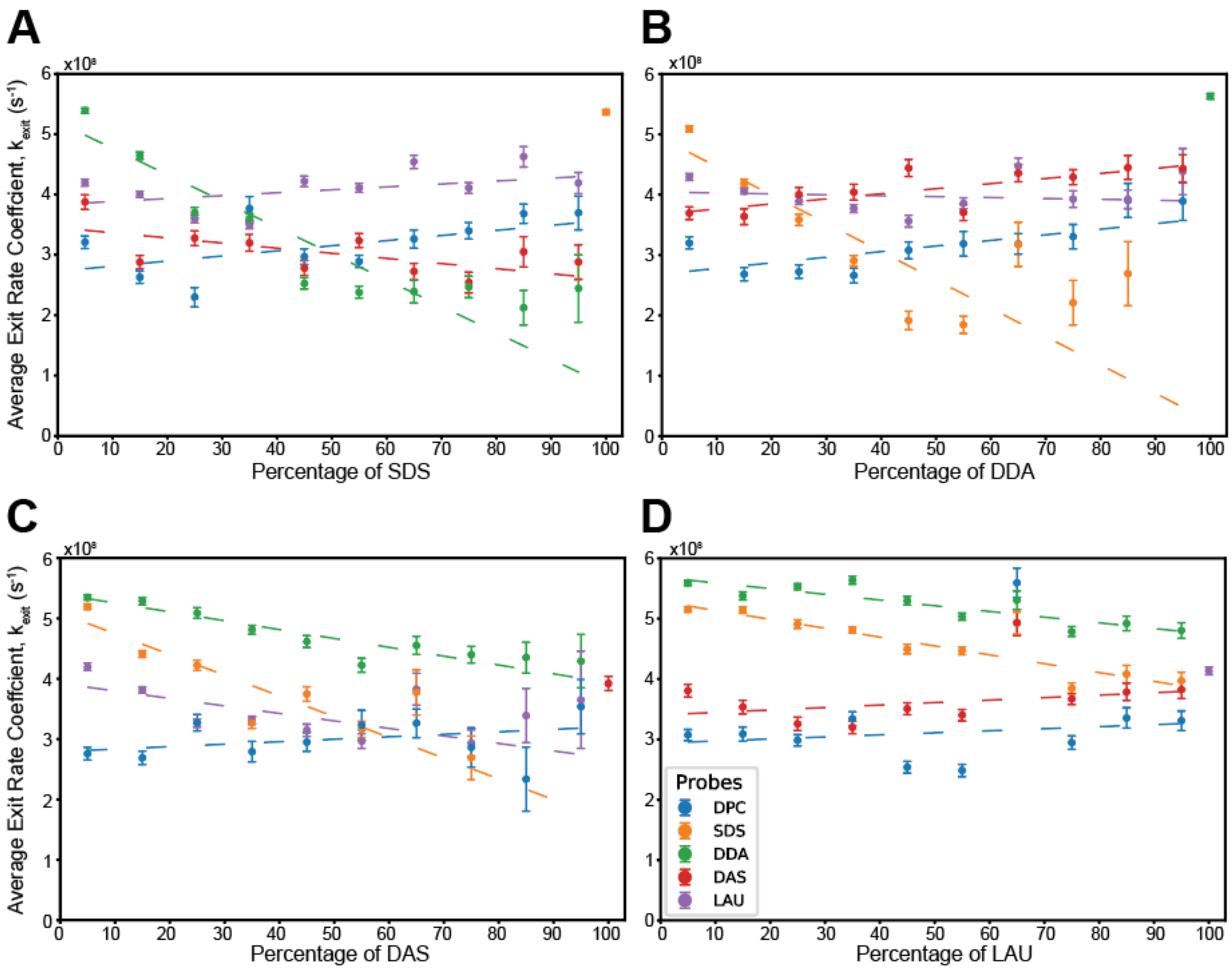

Supplementary Figure S2 - Exit reactions of monomeric lipids from pre-micellar clusters. A-D: average exit rate coefficients with varying cluster catalyst fractions. **A)** SDS as catalyst. R^2 values for probes: DPC – 0.301, DDA – 0.852, DAS – 0.371, LAU – 0.159. **B)** DDA as catalyst. R^2 values for probes: DPC – 0.462, SDS – 0.764, DAS – 0.564, LAU – 0.025. **C)** DAS as catalyst. R^2 values for probes: DPC – 0.151, SDS – 0.804, DDA – 0.869, LAU – 0.455. **D)** LAU as catalyst. R^2 values for probes: DPC – 0.025, SDS – 0.847, DDA – 0.771, DAS – 0.100.

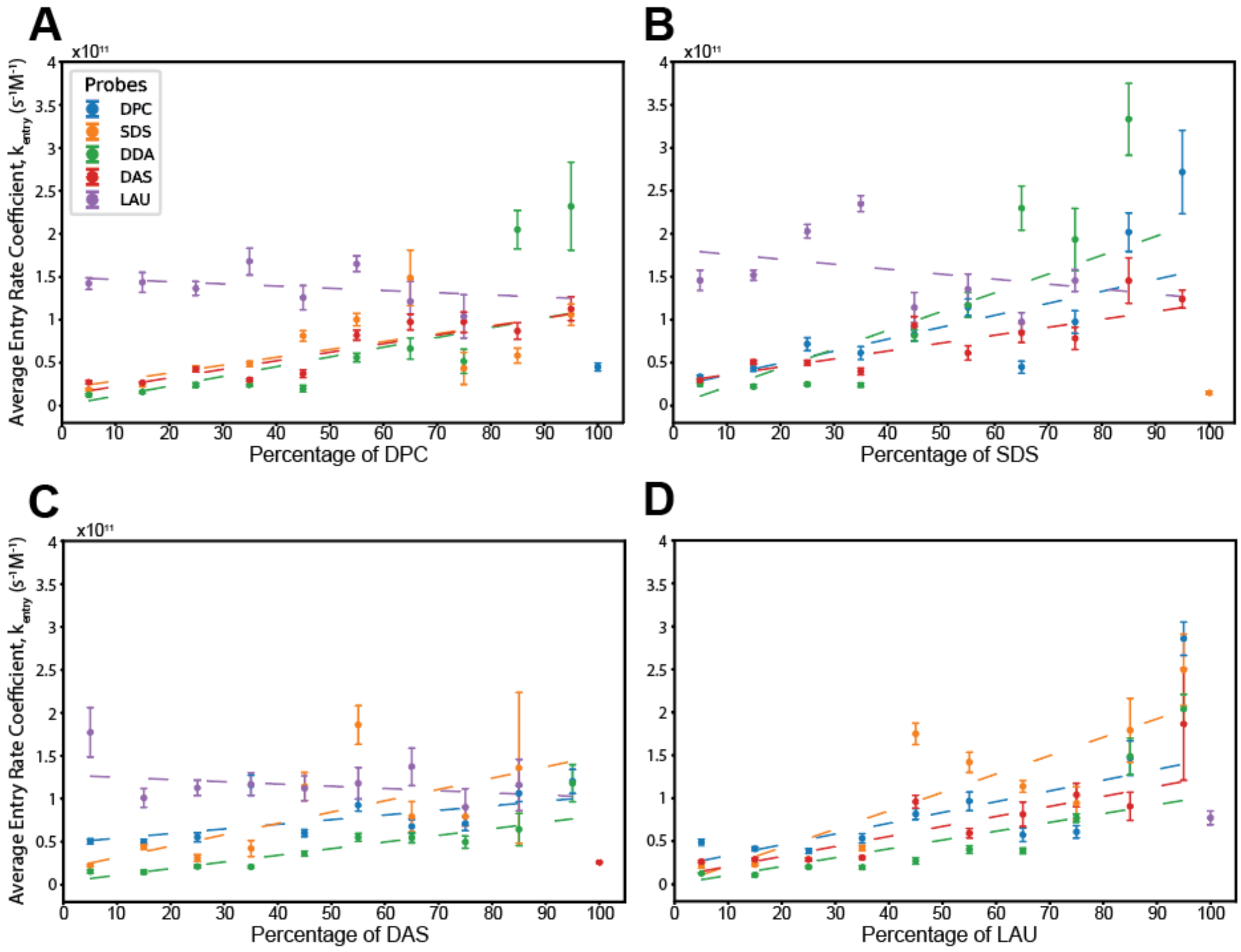

Supplementary Figure S3 – Entry reactions of monomeric lipids into pre-micellar clusters. A-D: average entry rate coefficients with varying cluster catalyst fractions. **A)** DPC as catalyst. R^2 values for probes: SDS – 0.488, DDA – 0.554, DAS – 0.809, LAU – 0.101. **B)** SDS as catalyst. R^2 values for probes: DPC – 0.561, DDA – 0.657, DAS – 0.665, LAU – 0.087. **C)** DAS as catalyst. R^2 values for probes: DPC – 0.342, SDS – 0.492, DDA – 0.809, LAU – 0.078. **D)** LAU as catalyst. R^2 values for probes: DPC – 0.465, SDS – 0.732, DDA – 0.603, DAS – 0.698.

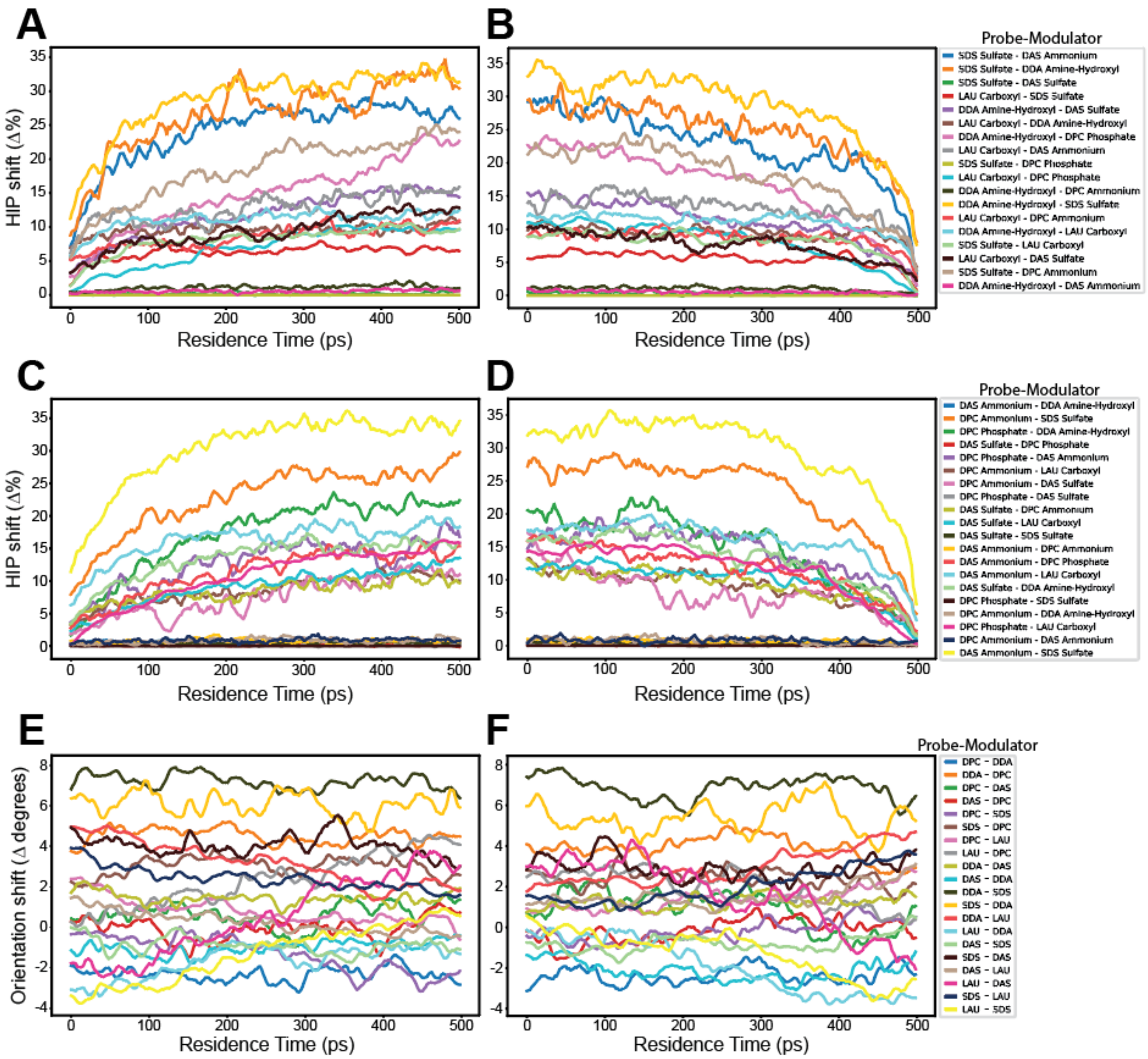

Supplementary Figure S4 – Specific headgroup interactions during lipid residence within clusters. **A-D)** Dynamic Headgroup Interaction Prevalence (HIP) plots depicting the shift in HIP values between catalyst-rich (50%-100%) and catalyst-poor (0%-50%) clusters along the first (A and C) and last (B and D) 0.5ns of the residence. Colors refer to distinct headgroup moieties interactions of probe-catalyst pairs. A and B feature single-moiety probes (SDS, DDA and LAU), while C and D feature double-moieties probes (DPC and DAS). **E-F)** Plots depicting shifts in probe orientation in relation to the geometrical center of the involved cluster, between catalyst-rich (50%-100%) and catalyst-poor (0%-50%) clusters along the first (E) and last (F) 0.5ns of the residence time. Colors refer to different probe-catalyst pairs.
